# Anatomy and Pathogenesis of Vascular Thoracic Outlet Syndrome

**DOI:** 10.7759/cureus.34470

**Published:** 2023-01-31

**Authors:** Miltiadis Perdikakis, Nikoleta Sinou, Stavros Angelis, George Tsakotos, Theodoros Mariolis-Sapsakos, Maria Piagkou, Dimitrios Filippou

**Affiliations:** 1 Anatomy, National and Kapodistrian University of Athens School of Medicine, Athens, GRC

**Keywords:** neurogenic, thoracic outlet syndrome, symptoms, anatomy, venous, arterial, vascular

## Abstract

The current literature review article describes the anatomy and pathogenesis of the vascular nature of thoracic outlet syndrome (TOS), as well as gathers the latest and most important information concerning its diagnostic methods and treatment. This syndrome’s subcategory includes the venous and the arterial. Data for this review was accumulated through the PubMed database in which only scientific studies published in the last decade (2012-2022) were searched. PubMed offered 347 results, of which 23 were judged suitable and used.

Non-invasive methods both for the diagnosis and the treatment of vascular TOS are gaining ground. Medicine, at this point, finds itself on the verge of slowly putting aside the previous invasive gold-standard methods, to be used only in the most urgent situations. The vascular thoracic outlet condition is a rare form of TOS but is also the most trouble-causing one and the deadliest. Fortunately, it can be more efficiently managed because of the current medical innovations. However, further research is needed to establish their already confirmed effectiveness, so they can be even more widely trusted and used.

## Introduction and background

Thoracic outlet syndrome (TOS) is characterized by neurological and vascular symptoms. The condition is caused by the compression of the subclavian vessels and brachial plexus (BP) as they exit the thoracic chest [1-3]. TOS is classified into three distinct forms, according to its pathophysiology: the neurogenic thoracic outlet syndrome (NTOS) after the BP compression, the arterial thoracic outlet syndrome (ATOS) after the subclavian artery (SCA) compression, and the venous thoracic outlet syndrome (VTOS) after the subclavian vein (SCV) compression [4]. Several studies have indicated NTOS as the most common type of TOS (94-95%) while ATOS and VTOS constitute only 5-6% [5].

The current review paper analyzes the detailed anatomy of the ATOS and the VTOS, their etiology, symptomatology, diagnosis, as well as their treatment.

## Review

Methodology and search strategy

**Figure 1:**
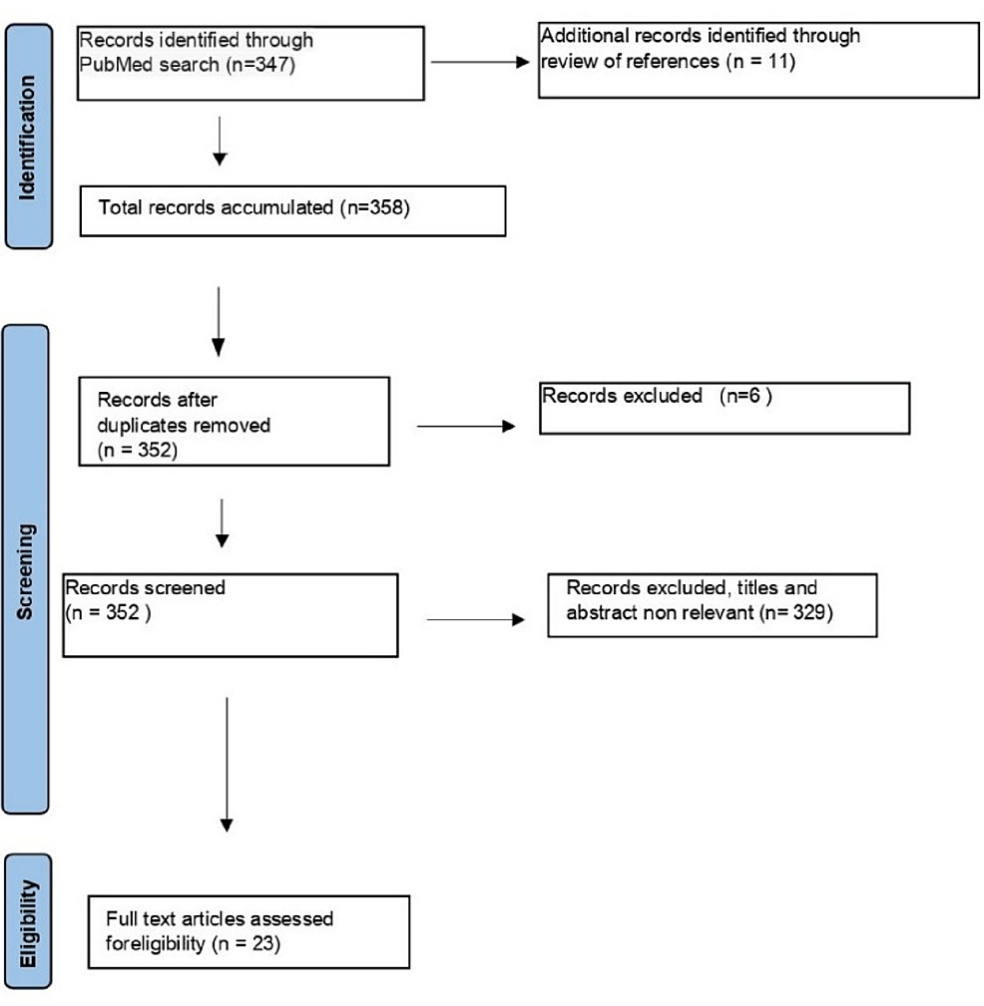
Diagram presenting the method based on which the articles for the paper were accumulated

This literature review is based on a search of articles in the PubMed database. Data were extracted using a common data extraction form. The information for this review was accumulated through the PubMed database in which "thoracic outlet syndrome" ([Title/Abstract]) AND ((ffrft[Filter]) AND (english[Filter])) was used as the search term and implemented as a Boolean scheme. A filter was also applied for articles only published during the latest decade (2012-2022) to be included in the search. The study was conducted in line with the PRISMA-ScR (Preferred Reporting Items for Systematic Reviews and Meta-Analyses extension for Scoping Reviews) guidelines. The identified records through the PubMed online database search were initially 347, and the additional ones through the references’ review were 11, thus there were 358 in total. So, the records after the duplicates’ removal, which were six, came to be 352. The full-text articles assessed for eligibility were 23, and the records excluded articles, titles, and abstracts not relevant were 329. Thus, 23 papers were finally included in this paper (Figure 1).

Literature review

Angiogenic TOS may occur after the SCA or SCV compression. These compressions mostly take place at some of the following anatomical spaces: 1. the superior pleural sinus, 2. the scalene triangle (between the anterior and middle scalene muscles), 3. the costoclavicular space (between the clavicle and first rib), and 4. the pectoralis minor space (between the pectoralis minor and subscapularis muscles) [6,7]. Moreover, the seventh cervical vertebra's (C7) ligaments and the first rib might be attached to the suprapleural membrane and compress the scalene triangle. The costoclavicular space surrounds the SCA, SCV, and BP and constitutes the most common anatomical space for venous TOS [1,4,8-10].

The thoracic outlet (TO) is known as the small space between the first thoracic vertebra (T1), the first rib, and the manubrium of the sternum. Specifically, the subclavius tendon borders with the SCV, which is separated from the SCA by the scalene. The BP is located at the posterior of the SCA [5]. The TO’s structure can easily be altered, thus causing a TOS pathology. TOS can be caused by a plethora of mechanisms, including trauma, anatomical alterations, and repetitive movements [4].

TO trauma can easily cause hemorrhage, hematoma, or an injured structure that may compress nerves, veins, or arteries [5]. TO trauma can also cause fibrosis [10]. This type of injury is accompanied by NTOS [6]. Moreover, repetitive motions can induce muscle hypertrophy. Hypertrophy leads to compression of the structure and, as a result, the BP, SCV, or SCA may be injured. The result is the same with traumatic events, but it is essential to emphasize that VTOS is the most possible outcome [6]. Finally, anatomic variants can incite TOS. Cervical or anomalous first ribs that are fused with the second rib can alter the TO dimensions, and therefore, make the space contract and extend. So, vessel and nerve injury can occur and cause TOS, usually ATOS [5,6].

The VTOS constitutes 3-4% of the incidents [6,7]. This form causes SCV compression and, therefore, thrombosis. The vein may often be inflamed, causing scarring, and narrowing over the years. Also, it may cause aching pain. Patients often observe dilated veins in the chest and back area. VTOS is often the result of repetitive motions and traumatic events. For these reasons, medical implants, such as catheters and pacemakers, are used to reduce the veins’ compression [5]. The ATOS constitutes only 1-2% of the occasions [10]. The continuous SCA friction and the pulsation of the underlying first rib may cause fibrosis, narrowing of the artery, aneurysms, and thrombosis. Therefore, the patients complain of pain during the arm’s activity, as well as after the occurrence of an embolization [5].

Symptoms that indicate a vascular TOS are not easily identified. ATOS is generally asymptomatic (aneurysm, occlusion, or silent embolization) and the symptoms begin to develop after embolization has occurred [11]. Because of the syndrome’s rarity, it is sometimes misdiagnosed [12]. General symptoms of vascular compression in the arm are paresthesia and numbness [13]. ATOS can be subcategorized based on the way of its pathogenesis in acute thrombosis, chronic stenosis, non-thrombotic ischemia, distal embolization, and total occlusion [14]. The main symptoms include claudication and pain during the arm’s activity, which slowly subsides after the end of the movement. In the acute phase, symptoms of ischemia are presented in the form of blue or white painful limbs [5]. If they remain untreated, they can lead to as far as gangrene [15]. Pain in the neck and arm is also usually present. Furthermore, TOS causes upper extremity pain because of the acute distal upper extremity ischemia, caused by the embolization [16]. A TOS can rarely present itself with non-limb-related consequences such as strokes [14].

VTOS (Paget-von Schroetter syndrome or effort thrombosis) is more common than ATOS. VTOS is usually prompted by repetitive arm movement and/or bone abnormalities [17]. VTOS is either acute or chronic. The first indication of the acute type is purple-red discoloration of the swollen extremity, and it can lead to visibly dilated superficial veins across the upper body [5]. It is generally presented with acute upper extremity swelling, cyanosis, and heaviness that ultimately produce pain [16]. Pallor and pulselessness of the affected upper extremity are also present [3]. Deep venous thrombosis and neurologic symptoms due to nerve compression are frequently developed [18]. An important complication of VTOS is pulmonary embolism (10-20%).

TOS diagnosis is complicated because of alternative disorders with similar manifestations. The correct diagnosis mostly depends on the familiarity of the physician with the TOS, as well as on the correct evaluation of the symptoms and the different risk factors of each patient. It consists of provocative physical exam maneuvers and radiographic and vascular studies [16]. Frequently, patients will present a mix of symptoms with concurrent NTOS, ATOS, and VTOS occurring simultaneously [14]. The diagnosis is made by confirming the compromised circulation of a vessel and identifying the anatomical factor that is responsible for the obstruction [15]. The clinical tests that assess the blood flow, such as provocative maneuvers, aid the clinician to determine the extent of the compression of the artery while imaging helps to define the anatomic source of the compression [16]. During the examination, any prior history of trauma should be noted. The pulse and blood pressure should be examined in both arms to detect possible differences between them (>20 mmHg). A true-positive provocative maneuver may provide suspicion of arterial dysfunction. The most usually practiced are Adson’s test and the overhead exercise test [15]. Chest radiography is insufficient to diagnose TOS. It can only evaluate bone abnormalities such as a cervical or dislocated first rib. Invasive angiography is the gold standard method of identifying VTOS, but it is sometimes not preferred because of its invasive nature [16]. It is usually reserved for intraprocedural interventional guidance. The performance of the arteriography with the patient seated provides bigger sensitivity than in other positions [15].

Duplex ultrasound (DUS) should be the initial test of choice for VTOS, with CT and MRI giving additional anatomic details for surgical planning [5]. CT is the most efficient in evaluating bone, and MRI soft tissue abnormalities. DUS is the most dominant noninvasive study both in VTOS and ATOS. The dampening of the waveform or marked decrease in the velocities, while the arm is abducted, can assure the diagnosis of VTOS. Concerning ATOS, pulse waveforms are again reduced; however, velocities will increase with stenosis or be absent in complete occlusion [5]. The ultrasound has the advantage of assessing dynamic blood flow during compression maneuvers, with a decrease in arterial diameter, changes in peak velocity, or reproducible symptoms considered to be diagnostic of ATOS [4].

TOS management is not standardized and is largely dependent on its etiology. The goal of the ATOS treatment is the restoration of distal blood flow. Asymptomatic patients with SCA compression with no evidence of arterial degeneration may be managed non-surgically due to a low risk of complications. Moreover, they should undergo an upper extremity DUS every six months. Surgical treatment is required for patients with symptoms and/or arterial complications [4]. Conservative therapy is successful in most cases. It consists of a combination of physical and pharmaceutical therapy, as well as a more general remodeling of the patient’s way of life [19]. Surgical intervention is indicated in the vascular forms of TOS that are usually resistant to conservative management [3]. Delays in the treatment of symptomatic ATOS may lead to a sympathetically maintained chronic pain syndrome [20]. In patients with continuing or progressing symptoms, surgical decompression may be used to treat a TOS. In cases of acute thrombosis, thrombolysis is sometimes necessary to counteract ischemia that constitutes a danger to a limb [14]. There are generally three main principles that should be followed intraoperatively: decompression, vascular resection, and distal revascularization. First, the surgeon deals with the structures compressing the artery. Surgical decompression is relatively safe and is unlikely to result in significant neurologic compromise [20]. Then, any potential sort of arterial or venous embolus is removed. The reconstruction of the artery is mainly conducted with either one of three methods: anastomosis, graft placement, or bypass with the brachial artery. The decompression is performed through a transaxillary or supraclavicular approach, with the last being more suitable for ATOS. The infraclavicular approach is less common. Open surgical revascularization is considered the gold standard for ATOS, however, some studies indicate that less invasive approaches are sometimes more suitable [4]. Some patients may present with acute limb ischemia, which needs to be addressed before fixing the ATOS [12]. The symptoms of ATOS have more favorable results after an operational treatment than those of NTOS, and they rarely manifest themselves again [21].

VTOS involves consideration of thrombolysis, decompression, and venoplasty in addition to anticoagulation treatment. In acute VTOS, catheter-directed thrombolysis has been reported to produce >90% clinical success. The chronic type benefits from surgical decompression and venoplasty of the SCV stenosis is considered to reduce the rethrombosis risk. In cases of intermittent obstruction without thrombus or significant stenosis, surgical decompression is enough [4]. The operative principles are similar to ATOS: any acute limb-threatening issues should be addressed first and the causative factors soon after [12]. The endovascular approach to subclavian vessels has become increasingly popular due to reduced morbidity and surgical complications [22]. First rib resection is generally performed routinely in VTOS patients in combination with scalenectomy. A few studies have examined the role of venous angioplasty and stenting before decompressing the thoracic outlet, but the results were suboptimal [4,23].

## Conclusions

The vascular thoracic outlet syndrome (TOS) is characterized by subclavian artery (SCA) and/or subclavian vein (SCV) compression. It is subcategorized into arterial TOS (ATOS) and/or venous TOS (VTOS). Neurogenic TOS (NTOS) constitutes the vast majority of TOS cases (94-95%), followed by VTOS (3-4%) and ATOS (1-2%). Compression sites include the superior pleural sinus, scalene triangle, costoclavicular space, and pectoralis minor space. TOS may be caused by several kinds of traumas, repetitive movements, or anatomical variants on the space between the first thoracic vertebra, first rib, and manubrium of the sternum. ATOS may be asymptomatic or presented with paresthesia and numbness in the chronic phase, ischemia, and pain in the acute phase. VTOS, in the acute phase, is presented with purple-red discoloration, swelling and cyanosis, and thrombosis and embolism. It includes physical exam maneuvers, radiographic, chest radiography, invasive angiography, arteriography, Duplex ultrasound, CT, and MRI. Finally, asymptomatic patients can be managed conservatively with physical therapy while symptomatic ones are usually managed surgically.

## References

[1] Hardy A, Pougès C, Wavreille G, Behal H, Demondion X, Lefebvre G (2019). Thoracic outlet syndrome: diagnostic accuracy of MRI. Orthop Traumatol Surg Res.

[2] Hu J, Biederman R, Kashyap K, Wilson JT, Farah V, Franco T, Nguyen V (2021). Duplex ultrasound in the evaluation of venous and arterial thoracic outlet syndrome. JRSM Open.

[3] Huang JH, Zager EL (2004). Thoracic outlet syndrome. Neurosurgery.

[4] Hussain MA, Aljabri B, Al-Omran M (2016). Vascular thoracic outlet syndrome. Semin Thorac Cardiovasc Surg.

[5] Freischlag J, Orion K (2014). Understanding thoracic outlet syndrome. Scientifica (Cairo).

[6] Atasoy E (2004). Thoracic outlet syndrome: anatomy. Hand Clin.

[7] Spartalis E, Spartalis M, Tsilimigras DI (2018). Extensive or partial first rib resection for thoracic outlet syndrome? The contribution of three-dimensional imaging to the preoperative planning and the postoperative evaluation. Clin Case Rep.

[8] Dahlstrom KA, Olinger AB (2012). Descriptive anatomy of the interscalene triangle and the costoclavicular space and their relationship to thoracic outlet syndrome: a study of 60 cadavers. J Manipulative Physiol Ther.

[9] Kaplan T, Comert A, Esmer AF (2018). The importance of costoclavicular space on possible compression of the subclavian artery in the thoracic outlet region: a radio-anatomical study. Interact Cardiovasc Thorac Surg.

[10] Li N, Dierks G, Vervaeke HE (2021). Thoracic outlet syndrome: a narrative review. J Clin Med.

[11] Foley JM, Finlayson H, Travlos A (2012). A review of thoracic outlet syndrome and the possible role of botulinum toxin in the treatment of this syndrome. Toxins (Basel).

[12] Nguyen LL, Soo Hoo AJ (2021). Evaluation and management of arterial thoracic outlet syndrome. Thorac Surg Clin.

[13] Wijeratna MD, Troupis JM, Bell SN (2014). The use of four-dimensional computed tomography to diagnose costoclavicular impingement causing thoracic outlet syndrome. Shoulder Elbow.

[14] Huang J, Lauer J, Zurkiya O (2021). Arterial thoracic outlet syndrome. Cardiovasc Diagn Ther.

[15] Artico M, Santarelli MT, Stevanato G (2022). The role of congenital malformations of the thoracic outlet in the development of the syndrome. Folia Morphol (Warsz).

[16] Jones MR, Prabhakar A, Viswanath O (2019). Thoracic outlet syndrome: a comprehensive review of pathophysiology, diagnosis, and treatment. Pain Ther.

[17] Oliveira I, Leal F, Santos L, Almeida Pinto J, Nogueira L, Mesquita M (2022). Venous thoracic outlet syndrome: when exercising may be discouraged. Clin Case Rep.

[18] Yuschak E, Haq F, Chase S (2019). A case of venous thoracic outlet syndrome: primary care review of physical exam provocative tests and osteopathic manipulative technique considerations. Cureus.

[19] Mitsos S, Patrini D, Velo S (2017). Arterial thoracic outlet syndrome treated successfully with totally endoscopic first rib resection. Case Rep Pulmonol.

[20] Nichols AW (2009). Diagnosis and management of thoracic outlet syndrome. Curr Sports Med Rep.

[21] Bae M, Lee CW, Chung SW, Choi J, Kim MS (2015). Bypass surgery in arterial thoracic outlet syndrome. Korean J Thorac Cardiovasc Surg.

[22] Archie MM, Gelabert HA (2019). Endovascular reconstruction of subclavian artery aneurysms in patients with arterial thoracic outlet syndrome. Ann Vasc Surg.

[23] Rinehardt EK, Scarborough JE, Bennett KM (2017). Current practice of thoracic outlet decompression surgery in the United States. J Vasc Surg.

